# Evaluation of social autonomy of schizophrenic patients

**DOI:** 10.1192/j.eurpsy.2023.2289

**Published:** 2023-07-19

**Authors:** M. Zbidi, W. Bouali, W. Haouari, M. Kacem, R. Bensoussia, L. Zarrouk

**Affiliations:** Psychiatry department, Taher sfar University hospital of mahdia tunisia, Mahdia, Tunisia

## Abstract

**Introduction:**

Schizophrenia is a debilitating and mutilating mental illness, generally progressing in a Chronic way . It is at the origin of a limitation of social autonomy and source of psychological distress .

**Objectives:**

evaluate the effective social autonomy of schizophrenic patients.

**Methods:**

it is a cross-sectional study carried out at the EPS psychiatry consultation in Mahdia during a period of 3 months, with schizophrenic patients meeting the DSM 5 criteria, having an age varying from 19 to 65 years and whose duration of evolution was at least of one year. Have been excluded those in a state of decompensation, presenting a severe organic disease or having a major cognitive impairment. Information was collected from patients and fromtheir medical records using a pre-established questionnaire. The scale of social autonomy (EAS) of Legay with 17 items grouped into 5 dimensions was used for the evaluation.

**Results:**

The general characteristics of the 360 schizophrenic patients who met the inclusion criteria, revealed an average age of 40.2 years, a sex ratio of 2.33, a majority of single (55.8%), a low level of education(66.7%), an absence of professional activity (67.3%) and a deteriorated socioeconomic level (68.6%).Clinical Characteristics noted an average onset age of the disorder of 26 years, an average duration of evolution of 14 years and a preponderance of the residual type and of the episodic evolutionary course with residual symptoms between episodes respectively in 40.6 and 76.4%. The average of EAS scores were 39.08. Three quarters of the population (75.7%) had a score below 59. 24.3% of patients had scores between 60 and 108 signifying impaired social autonomy.

**Conclusions:**

The evaluation of effective social autonomy is essential for any therapeutic project considering psychosocial integration and rehabilitation of schizophrenic patients.

**Disclosure of Interest:**

None Declared

